# Anti-Obesity and Anti-Inflammatory Effects of Novel Carvacrol Derivatives on 3T3-L1 and WJ-MSCs Cells

**DOI:** 10.3390/ph16030340

**Published:** 2023-02-22

**Authors:** Ivana Cacciatore, Sonia Spalletta, Annalisa Di Rienzo, Vincenzo Flati, Erika Fornasari, Laura Pierdomenico, Piero Del Boccio, Silvia Valentinuzzi, Erica Costantini, Elena Toniato, Stefano Martinotti, Carmela Conte, Antonio Di Stefano, Iole Robuffo

**Affiliations:** 1 Department of Pharmacy, “G. d’Annunzio” University of Chieti-Pescara, 66100 Chieti, Italy; 2 Department of Medical, Oral and Biotechnological Sciences, “G. d’Annunzio” University of Chieti-Pescara, 66100 Chieti, Italy; 3 Department of Biotechnological and Applied Clinical Sciences, University of L’Aquila, 67100 L’Aquila, Italy; 4 Department of Medicine and Aging Sciences, Center on Advanced Studies and Technologies (CAST), “G. d’Annunzio” University of Chieti-Pescara, 66100 Chieti, Italy; 5 Center for Advanced Studies and Technology (CAST), University “G. d’Annunzio” of Chieti-Pescara, Via dei Vestini 31, 66100 Chieti, Italy; 6 Department of Medicine and Aging Sciences, “G. d’Annunzio” University of Chieti-Pescara, 66100 Chieti, Italy; 7 Department of Innovative Technology in Medicine and Odontoiatrics, “G. d’Annunzio” University of Chieti-Pescara, 66100 Chieti, Italy; 8 Center for Advanced Studies and Technology (CAST), “G. d’Annunzio” University of Chieti-Pescara, 66100 Chieti, Italy; 9 Department of Pharmaceutical Sciences, University of Perugia, 06123 Perugia, Italy; 10 Institute of Molecular Genetics “Luigi Luca Cavalli Sforza”, National Research Council, Section of Chieti, 66100 Chieti, Italy

**Keywords:** adipogenesis, anti-inflammatory drugs, carvacrol, obesity

## Abstract

(1) Background: Obesity, a complex metabolic disease resulting from an imbalance between food consumption and energy expenditure, leads to an increase in adipocytes and chronic inflammatory conditions. The aim of this paper was to synthesize a small series of carvacrol derivatives (**CD1-3**) that are able to reduce both adipogenesis and the inflammatory status often associated with the progression of the obesity disease. (2) Methods: The synthesis of **CD1-3** was performed using classical procedures in a solution phase. Biological studies were performed on three cell lines: 3T3-L1, WJ-MSCs, and THP-1. The anti-adipogenic properties of **CD1-3** were evaluated using western blotting and densitometric analysis by assessing the expression of obesity-related proteins, such as ChREBP. The anti-inflammatory effect was estimated by measuring the reduction in TNF-α expression in **CD1-3**-treated THP-1 cells. (3) Results: **CD1-3**—obtained through a direct linkage between the carboxylic moiety of anti-inflammatory drugs (Ibuprofen, Flurbiprofen, and Naproxen) and the hydroxyl group of carvacrol—have an inhibitory effect on the accumulation of lipids in both 3T3-L1 and WJ-MSCs cell cultures and an anti-inflammatory effect by reducing TNF- α levels in THP-1 cells. (4) Conclusions: Considering the physicochemical properties, stability, and biological data, the **CD3** derivative—obtained by a direct linkage between carvacrol and naproxen—resulted in the best candidate, displaying anti-obesity and anti-inflammatory effects in vitro.

## 1. Introduction

Obesity, one of the global issues for public health, plays a central role in the onset of the metabolic syndrome and its implications [1,2,3,4]. It results from an imbalance between energy intake and energy expenditure and leads to hyperplasia and adipocyte hypertrophy [5]. It is associated with chronic inflammatory conditions, an increase in cytokines release, and inflammatory process activation [6,7]. Tumor Necrosis Factor-alpha (TNF-α), produced by both lymphoid cells and non-lymphoid cells, is the key regulator in the inflammatory process initiation and potentiation, and its levels are elevated in adipose tissues [8]. Moreover, TNF-α induces Cyclooxygenase-2 (COX-2) expression [9]. The role of TNF-α in obesity-related insulin resistance has been recently suggested. Furthermore, in the adipose tissue of several obesity models, TNF-α expression levels are high. The neutralization of TNF-α in experimental models improves insulin sensitivity in muscle and adipose tissue via the insulin tyrosine kinase receptor pathway [10].

Non-steroidal anti-inflammatory drugs (NSAIDs) have anti-inflammatory, analgesic, and antipyretic activities. Their mechanism of action is due to both the inhibition of cyclooxygenases (COX-1 and COX-2) [11] and the modulation of peroxisome proliferator-activated receptors (PPAR)—specifically, PPAR-γ, a central adipogenic regulator [12]. High doses of NSAIDs can modulate PPAR activation in vitro [13] and up-regulate the adipogenic differentiation process. Notably, indomethacin and ibuprofen can induce adipocyte differentiation in C3H10T1/2 cells, 3T3-L1, and hepatocytes (both in vitro and in vivo) by activating the PPAR-y receptor [14,15]. Ibuprofen and naproxen stimulate adipogenesis by activating PPAR isoforms and inhibiting prostaglandin H(2) synthases [16,17]. Flurbiprofen is another cyclooxygenases inhibitor, but unlike other NSAIDs, it has an anti-obesity effect [18]. It belongs to the class of drugs for the treatment of obesity due to its ability to reduce the accumulation of unfolded proteins, which causes endoplasmic reticulum stress, which is responsible for obesity development. Its anti-obesity properties are due to its capacity to attenuate leptin resistance and the subsequent amelioration of glucose deprivation-induced leptin resistance, which is involved in the pathophysiology of obesity [19]. NSAIDs, however, have a lot of adverse effects: enhanced gut permeability and inflammation, anemia, malabsorption, and mucosal ulceration. Their prolonged use increases the risk of gastrointestinal bleeding, ulcerations, perforations, and cardiovascular and cerebrovascular effects [20,21].

Many natural products contained in foods, such as carvacrol ([2-methyl-5-(1-methyl-ethyl)phenol] or Car), can reduce adipogenesis [22,23,24,25]. It is a mono-terpenoid present in essential oils of aromatic plants, such as oregano and thyme. It inhibits obesity in HFD-fed mice by diminishing body weight and visceral fat-pad weights and reducing plasma lipid levels [26]. Car lowers the levels of pro-inflammatory cytokines in adipose tissues by impeding toll-like receptor 2 (TLR2)- and TLR4-mediated signaling [27,28,29]. It has also been demonstrated that Car possesses a wide range of properties, including antimicrobial and anti-inflammatory activity [30]. In the last decade, medicinal chemistry-based approaches (prodrugs, codrugs, derivatives, and/or hybrids) were also used by us to improve the efficacy and pharmacokinetic properties of Car [31,32,33,34,35]. Using these strategies, Car was linked to amino acids (to improve water solubility) [31], cinnamic acid derivatives (to obtain topic drugs for the treatment of skin infections) [32], antioxidants (to enhance its potency as an antimicrobial and antifungal drug) [33], and benzyl moiety, especially meta- or para-substituted (to obtain dual inhibitors of *H. pylori* strains and AGS cell proliferation) [34]. In this context, considering the wide potential of this terpenoid as a pharmaceutical compound, the aim of this work was to develop a small series of Car derivatives (**CD1-3**) with anti-adipogenic and anti-inflammatory properties endowed with reduced side effects typical of NSAIDs. Car was chosen as a moiety to link, by an ester linkage, to ibuprofen (**CD1**), flurbiprofen (**CD2**), and naproxen (**CD3**) for several reasons: (a) to reduce the adipogenic effects that characterize the selected NSAIDs; (b) to keep the anti-inflammatory efficacy of the novel derivatives inalterable; (c) to mask the carboxylic functions of the selected NSAIDs that are correlated with gastrointestinal toxicity; and (d) to obtain no toxic derivatives by the introduction of Car, since it is considered GRAS (Generally Recognized As Safe) and approved for food use. Particularly, in the present study, the physicochemical properties, stability profiles, and biological evaluation of **CD1-3** on undifferentiated and differentiated 3T3-L1 and Wj1-MSCs cells were reported. The anti-inflammatory profile was assessed on THP1-cells, evaluating the TNF-α release after treatment with **CD1-3**.

## 2. Results and Discussion

### 2.1. Synthesis

**Scheme 1:**
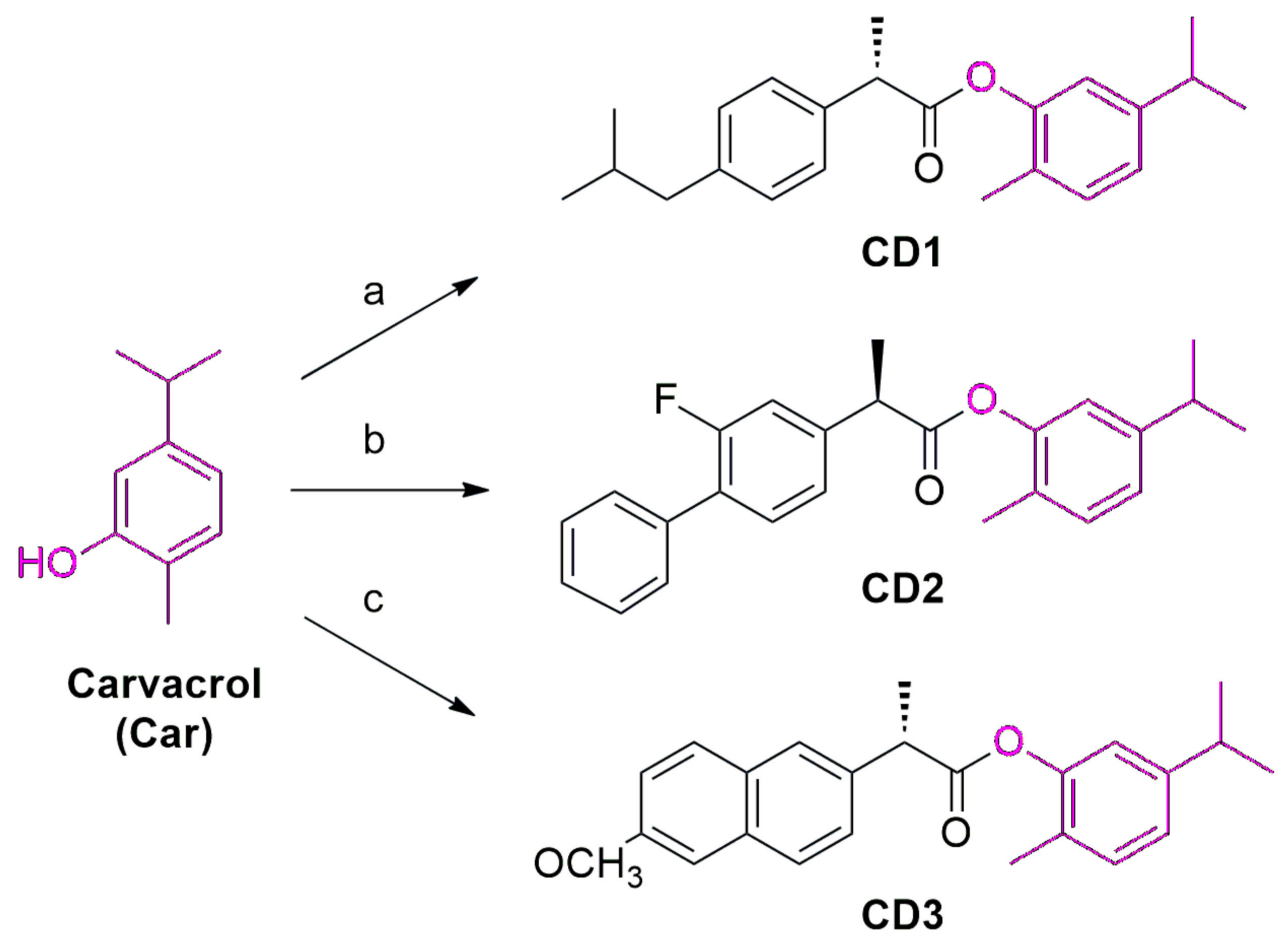
Experimental conditions for the synthesis of **CD1-3**: (**a**) ibuprofen, BOP, TEA, and dry DCM/DMF (1:1) at 0 °C for 30′ and then at 40 °C for 4 h; (**b**) flurbiprofen, DCC, DMAP, and dry THF for 24 h at rt; (**c**) naproxen, DCC, DMAP, and dry THF for 24 h at rt.

The syntheses of **CD1-3** are shown in Scheme 1. As reported by de Oliveira Pedrosa Rolim et al. [35], (S)-5-isopropyl-2-methylphenyl 2-(4-isobutilphenyl) propanoate (**CD1**) was obtained by the esterification of the carboxyl group of (R,S)-2-[4-(2-methylpropyl)phenyl]-propanoic acid with the -OH of Car, in the presence of benzotriazol-1-yloxytri(dimethylamine) hexafluorophosphate (BOP) as a coupling agent. (R)-5-isopropyl-2-methyl-phenyl-2-(6-methoxynaphthalen-2-yl) propanoate (**CD2**) and (S)-5-isopropyl-2-methyl-phenyl-2-(6-methoxynaphthalen-2-yl) propanoate (**CD3**) were prepared through the conjugation of the appropriate carboxyl acid, 2-(2-fluoro-[1,1′-biphenyl]-4-yl) propanoic acid (Flurbiprofen) or 2-(6-methoxynaphthalen-2-yl)propanoic acid (Naproxen), respectively, and Car by using N,N′-Dicyclohexylcarbodiimide (DCC) and 4-dimethylaminopyridine (DMAP).

The structures of **CD1-3** were verified by ^1^H- and ^13^C-NMR and HR-MS spectra (NMR and MS spectra are reported in the Supporting Information). The purity of **CD1-3** was >97%, as stated by HPLC chromatograms (Supporting Information). 

### 2.2. In Silico Evaluation of Physicochemical Properties

The SwissADME software was used to predict pharmacokinetic and physicochemical properties, such as water solubility, Lipinski’s rule parameters, topological polar surface area (TPSA), molar refractivity (MR), and Rotable Bonds (RB) [36]. Lipinski’s rule of five includes some properties, such as the water/octanol partition coefficient (log P), molecular weight (MW), Hydrogen Bond Acceptors (HBA), and Hydrogen Bond Donors (HBD). A compound is considered to possess good absorption and permeability when characterized by an MW < 500, an HBD < 5, a log *p* value < 5, and an HBA < 10. According to Table 1, **CD1-3**, even if they have a LogP slightly higher than 5, possess good physicochemical properties that are necessary to develop a drug for oral administration.

### 2.3. Stability Studies

Hydrolysis studies of **CD1-3** were achieved at 37 °C in simulated gastric fluid (hydrochloric acid buffer, pH 1.2), phosphate buffer (pH 7.4), and human plasma (Table 2) [37]. All derivatives were more stable in an acidic environment (pH 1.2) than in physiological conditions (pH 7.4). As we expected, the presence of the ester linkage resulted in more susceptibility to the basic environments compared to the acidic one. Additionally, in plasma fluid, **CD1-3** were very stable, displaying a t_1/2_ greater than 2.5 h, which would ensure the achievement of the target site, following oral administration and avoiding the degradation. **CD3** resulted in the derivative endowed with the best physicochemical properties, being more water soluble, less lipophilic, and more stable in human plasma compared to **CD1-2** (Table 1 and Table 2). These data confirmed that the presence of 2-methoxynaphthalene moiety as a scaffold in **CD3** ensures an elevated stability against the proteolysis due to its steric hindrance compared to the isobutyl benzene or fluoro-biphenyl structures present in **CD1** and **CD2**, respectively.

### 2.4. Anti-Adipogenic Activity

3T3-L1 and WJ-MSCs cells were chosen as experimental models to evaluate the in vitro inhibitory effects of **CD1-3** on lipid accumulation and assess the expression of anti-obesity-related proteins following **CD1-3** treatment. Following our previous work, the Oil Red O staining was used to assess the cell differentiation into mature adipocytes (Figures S1 and S2), and both cell lines were grown in an adipogenic differentiation medium and treated with 25 μM Car [27]. Full differentiation was completed following 7 days in 3T3-L1 cells (Figure S1) and following 17 days in WJ-MSCs cells (Figure S2). As we previously reported [27], at 5 days of differentiation, Car has no effect on 3T3-L1 differentiation, while it reduced their differentiation by 40% at 7 days, suggesting that it drastically reduces adipogenic differentiation in 3T3-L1 cells. Similarly, Car (25 μM) reduced the differentiation in two subcultures of Wharton’s jelly WJ-MSCs, WJ-1 and WJ-2, albeit to a lesser extent than 3T3-L1 cells (30% vs. 50%), due to the toxicity-induced mortality of Car in WJ-MSCs cells (Figure S2).

**Figure 1:**
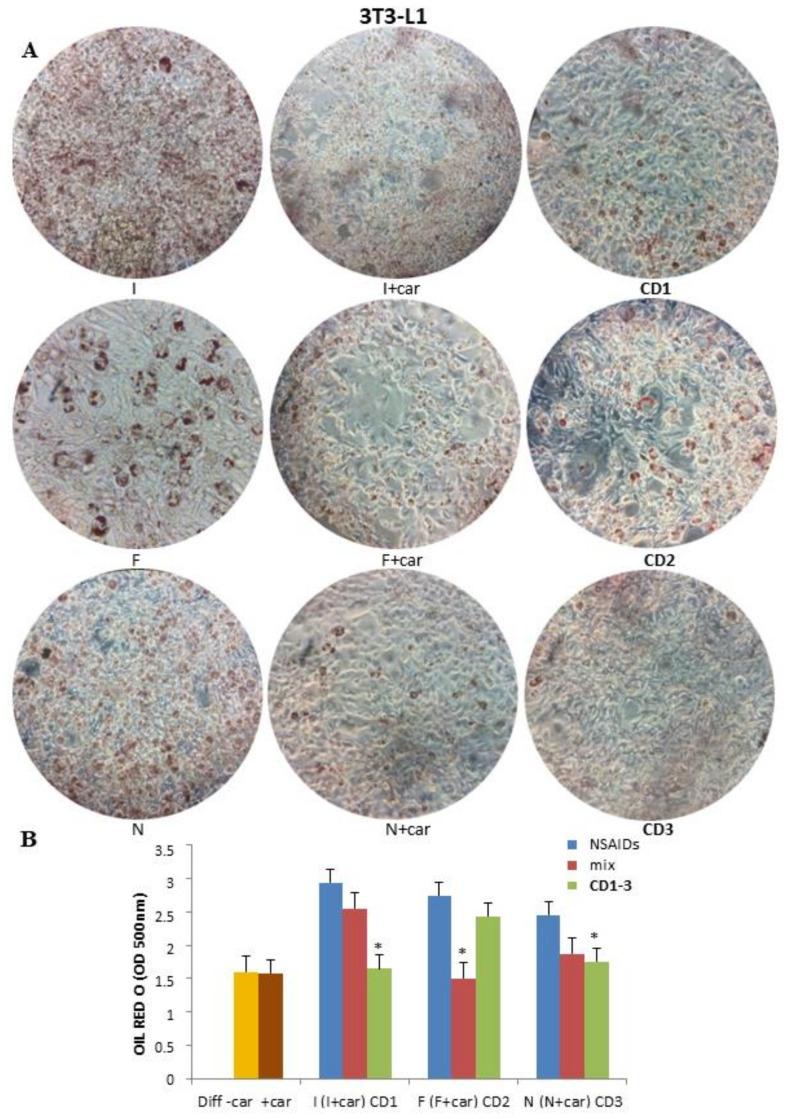
Effect of **CD1-3** on adipocyte differentiation in 3T3-L1 cells. (**A**) 3T3-L1 preadipocytes were cultivated in an adipogenic differentiation medium and treated with NSAIDs alone, a physical mixture of selected NSAID and 25 μM Car, and **CD1-3**. (**B**) The visualization of triglyceride accumulation was conducted by Oil Red O staining. Lipid accumulation was observed and measured at 5 days of differentiation by spectrophotometry. Data are produced as the mean ± SD (*n* = 3). * *p* < 0.05: treated cells vs. control (diff − Car) and Car (diff + Car).

After the differentiation, both cell lines were treated with the derivatives **CD1-3** and a physical mixture of the selected NSAID (Ibuprofen (I), Flurbiprofen (F), and Naproxen (N)) and 25 μM Car (in equimolar ratio) to verify if the combination of the two pharmacophores into a single molecule had a synergic effect compared to the single molecules. As shown in Figure 1, ibuprofen, flurbiprofen, and naproxen showed a strong adipogenic action compared to the control (diff − Car). In fact, the 3T3-L1 cells treated with the selected NSAIDs anticipated the total differentiation at 5 days of differentiation, which is comparable to the 7 days of the control. In the presence of the physical mixture of NSAIDs plus Car (25 μM), the adipogenic activity of the NSAIDs continued, but it was slightly slowed down by Car. On the other hand, **CD1-3** demonstrated a drastic reduction in adipogenesis, which was comparable to that of Car alone; notably, **CD1** and **CD3**, containing ibuprofen and naproxen, respectively, showed the highest antiadipogenic activity compared to **CD2**. 

**Figure 2:**
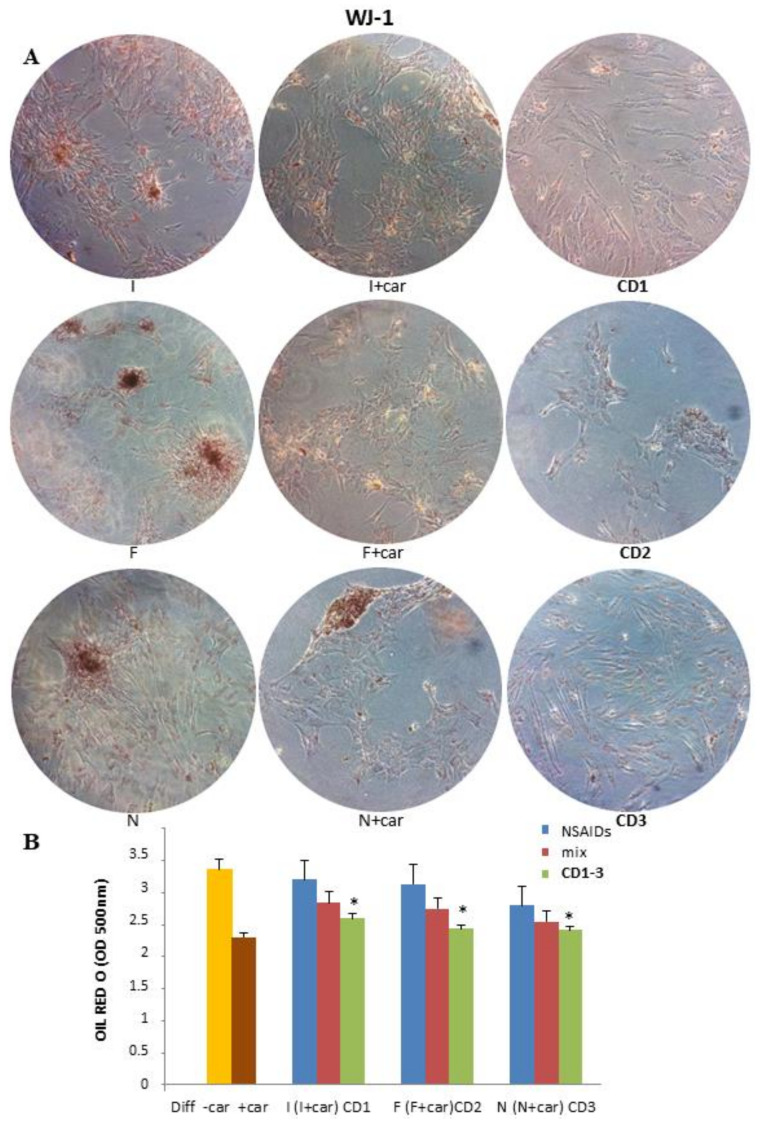
Effect of **CD1-3** on adipocyte differentiation in subculture Wj1-MSCs cells. (**A**) The subculture WJ-1 was grown in an adipogenic differentiation medium until total differentiation (17 days) and treated with NSAIDs alone, a physical mixture of selected NSAID and 25 μM Car, and **CD1-3**. (**B**) The visualization of triglyceride accumulation was conducted by Oil Red O staining. Lipid accumulation was measured by spectrophotometry. Data are produced as the mean ± SD (*n* = 3). * *p* < 0.05: treated cells vs. control (diff − Car) and Car (diff + Car).

At the same time, the subculture WJ-1 was grown in an adipogenic differentiation medium until total differentiation (17 days) and treated with NSAIDs alone, a physical mixture of the selected NSAID and 25 μM Car, and **CD1-3**. As shown in Figure 2, ibuprofen, flurbiprofen, and naproxen showed an adipogenic action compared with the control (diff − Car). Compared to 3T3-L1 cells, both the physical mixtures (selected NSAID and Car) and **CD1-3** reduced adipogenesis.

#### 2.4.1. Effect of CD1–3 on ChREBP Activity during Adipogenic Differentiation

Adipogenesis is a permanent process in adult adipose tissues during which preadipocytes, if subjected to appropriate stimuli, can proliferate and differentiate [38]. The transcription factor carbohydrate response element binding protein (ChREBP) is the main regulator of the adipogenic differentiation process [39]. 

**Figure 3:**
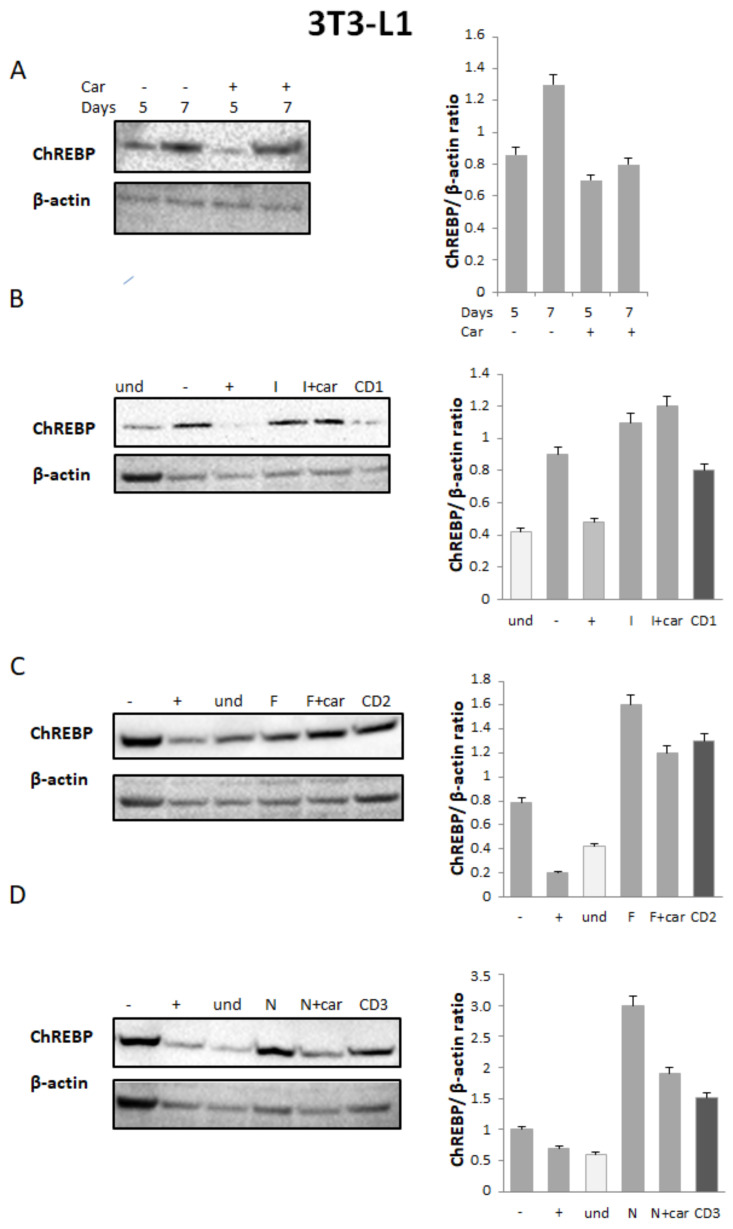
Effect of **CD1-3** on ChREBP activity during the adipogenic process. (**A**) Western blot and densitometric analysis of ChREBP in 3T3 cells undergoing adipogenic differentiation for, respectively, 5 to 7 days without (diff − Car) or with (diff + Car) 25 μM Car; (**B**–**D**) Western blot and densitometric analysis of ChREBP in 3T3-L1 cells undergoing adipogenic differentiation for 7 days without (−Car) or with (+Car) 25 μM Car; NSAID alone (I = Ibuprofen, F = Flurbiprofen, N = Naproxen); a physical mixture of selected NSAID and 25 μM Car; and **CD1-3**. Anti-β-actin was used as the protein loading control. Bars depict the means ± SE. (*n* = 2).

Since the transcription factor, ChREBP, is the major factor responsible for de novo lipogenesis, we analyzed its modulation by NSAIDs alone, a physical mixture of the selected NSAID and 25 μM Car, and **CD1-3** in 3T3-L1 cells. Western blot and densitometric analysis showed that ChREBP protein levels increased during adipogenic differentiation, whereas they were reduced by both Car and **CD1-3** (Figure 3). The maximum expression of ChREBP was at 7 days of differentiation (Figure 3A), which was comparable to NSAIDs (Figure 3B–D). The highest reduction in ChREBP protein levels was obtained after treatment with **CD3** (Figure 3D). It has been reported that the reduction in ChREBP expression levels by various anti-adipogenic compounds is a valid therapeutic method for counteracting obesity, so **CD3** possessing this property could be proposed for a deepened investigation.

#### 2.4.2. Effect of CD1–3 on TNF-α Release in LPS-Treated THP-1 Cells

The chronic systemic low-grade inflammation of white adipose tissue is associated with obesity, which can lead to metabolic syndrome and insulin resistance. The inflammatory process is triggered by the synthesis and secretion of pro-inflammatory cytokines in response to an inflammatory insult. The irregular secretion of adipokines stimulates the recruitment and invasion of macrophages to the adipose tissue and increases levels of proinflammatory cytokines. In particular, TNF-α is known as an adipokine overexpressed in the human adipose and muscle tissues of overweight people, with a crucial role in mediating insulin resistance [40].

To evaluate the anti-inflammatory effect of **CD1-3**, THP-1 cells were used [41]. These cells derive from the peripheral blood of a childhood case of acute monocytic leukemia and are used as a model for human monocytes. Indeed, when stimulated by phorbol myristate acetate (PMA), they can differentiate into macrophage-like cells resembling the properties of mature macrophages (N). 

**Figure 4:**
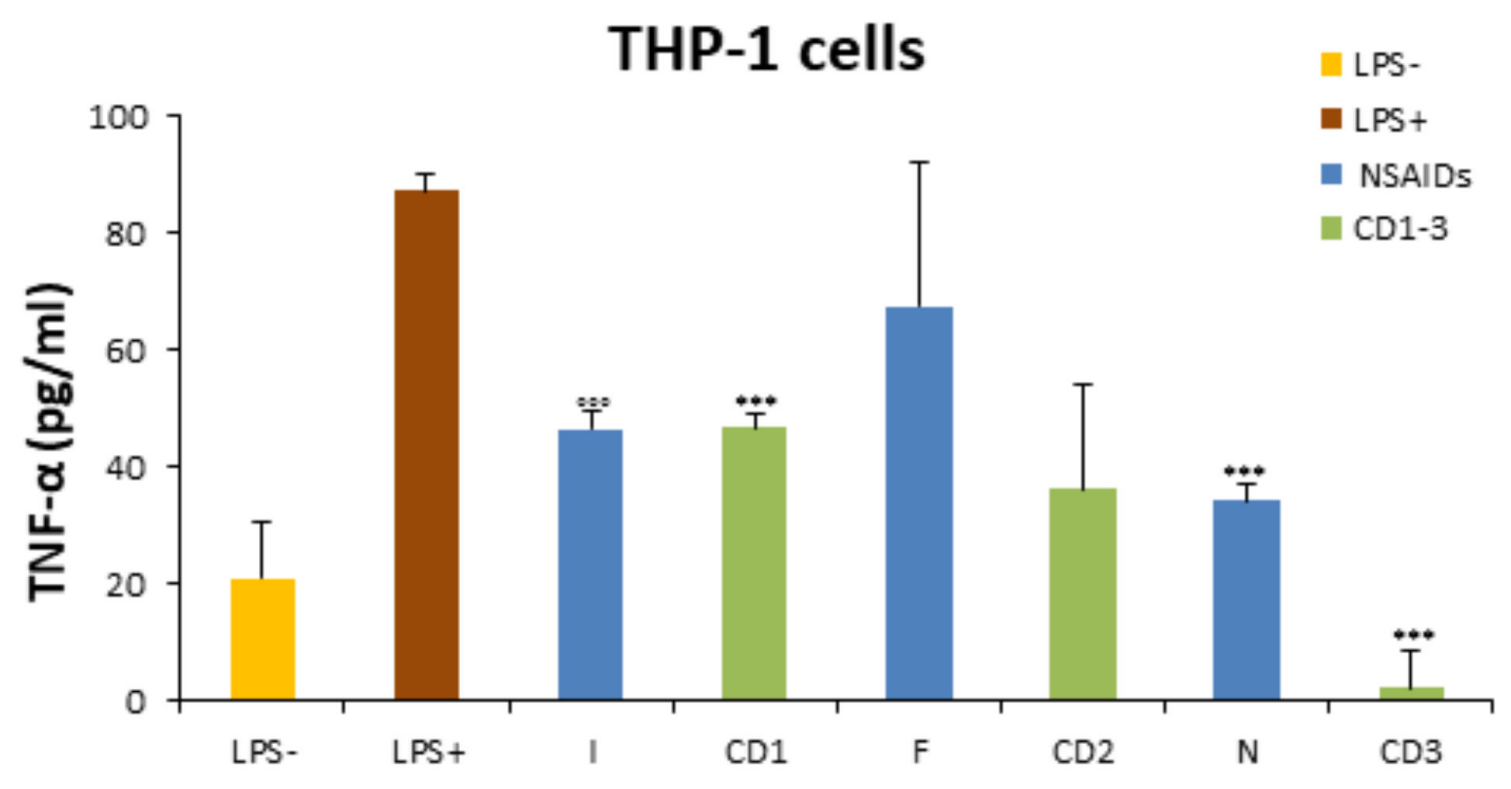
Reduction in TNF-α levels by **CD1-3** in THP1-cells. TNF-α secretion was determined by ELISA assay in LPS-treated-THP-1 cells (100 ng/mL), NSAID (I = Ibuprofen, F = Flurbiprofen, N = Naproxen), or **CD1-3** derivates (25 μM) for 24 h. All values are reported as the mean ± SD. °°° *p* < 0.001 compared with the LPS^−^ group, *** *p* < 0.001 compared with the LPS^+^ group.

The potential anti-inflammatory effect of **CD1-3** on THP-1-derived macrophages through the release of TNF-α levels was analyzed by the ELISA assay. To obtain an in vitro inflammatory experimental model, THP-1 cells were stimulated with LPS, a major inducer of the production and release of TNF-α [42]. THP-1 cells were also treated with NSAIDs. We showed that, compared with untreated cells, TNF-α production was remarkably increased after LPS stimulation for 24 h (TNF-α = 87.20 ± 2.605 pg/mL), while the treatment with **CD1-3** for 24 h led to a reduction in TNF-α levels with respect to the LPS-treated cells (**CD1** = 46.70 ± 2.246 pg/mL; **CD2** = 36.14 ± 17.791 pg/mL) (Figure 4). Interestingly, THP-1 cells showed a drastic decrease in TNF-α levels when treated with **CD3** (2.0 ± 6.595 pg/mL). Statistical significance was reported in comparison to both LPS-treated cells and untreated cells (Figure 4). 

In this study, based on the clue that Car, one of the main components of oregano and thyme, displayed an anti-adipogenic activity, we screened a small series of Car derivatives to discover more effective drugs endowed with both anti-inflammatory properties and reduced adipogenic activities. The literature data report a strong correlation between obesity and several pathological complications such as cardiovascular or renal dysfunctions, diabetes, and oxidative stress [43,44,45,46]. Notably, obesity is often also associated with the pro-inflammatory state that characterizes the above-cited pathologies [47]. The enhancement of proinflammatory cytokines (IL-6, TNF-α, and IL-1β) and an imbalance of adipokines are involved in the development and growth of the adipose tissue. Recent findings report that some phytochemicals are able to inhibit biochemical markers related to adipogenesis and inflammation. Extracts of S. coreana Nakai, a bamboo tree of South Corea, are endowed with both anti-adipogenic activities by avoiding macrophage chemotaxis and anti-inflammatory properties by inhibiting the NF-kB pathway [48]. The same properties are possessed by the extract of rhizomes of A. helferiana or of the Ligularia taquetii Nakai [49,50].

In the literature, there are several examples of natural molecules with dual functions, but they are not commercially available since their safety and efficacy have yet to be deeply studied. At the same time, it is well known that the prolonged treatment of inflammatory conditions using classical NSAIDs can favor the development of the adipogenesis process. Despite a considerable number of natural compounds, few examples of synthetic molecules as dual-acting (anti-inflammatory and anti-adipogenic activities) drugs are reported in the literature [51,52]. In fact, it is rare to find synthetic derivatives with both activities. Starting from these considerations, **CD1-3** were developed as novel resources in the development of anti-inflammatory and anti-obesity drugs.

Gathering together all the obtained data, we can assume that the **CD1-3**, obtained through a direct linkage between Car and NSAIDs, resulted in molecules endowed with good drug-like properties and quite good stability for an eventual oral administration. An ester linkage was introduced in all derivatives, but the derivative endowed with the best physicochemical properties resulted in **CD3** being more water soluble, less lipophilic, and more stable in human plasma compared to **CD1-2**. These data proved that the presence of the 2-methoxynaphthalene moiety as a scaffold in **CD3** could ensure great stability against the proteolysis due to its steric hindrance compared to the isobutyl benzene or fluoro-biphenyl moieties present in **CD1** and **CD2**, respectively. 

As already reported in the literature, we observed that ibuprofen, naproxen, and flurbiprofen stimulate adipogenesis in both adipogenic cell models, murine 3T3-L1 and human WJ–MSCs. In prolonged therapies with NSAIDs, among their side effects, there is the stimulation of preadipocytes, which leads to the formation of new adipose tissue, which is involved in the development of various pathologies. Our derivative—in particular, **CD3**—is able to reduce adipogenesis and, at the same time, possesses an anti-inflammatory effect due to the presence of naproxen. This molecule could reduce the formation of novel adipose tissue, so it could be beneficial for long anti-inflammatory therapies.

## 3. Materials and Methods

All of the reagents were supplied by Sigma-Aldrich Co. (St. Louis, MO, USA). Chromatographic columns (Merck 60, 230–400 mesh ASTM silica gel) were used for purifications. NMR spectra were recorded with a Varian VXR-300 spectrometer (Varian Medical Systems, Inc., Paolo Alto, CA, USA). The analyses suggested by the symbols of the elements were within ± 0.4% of the theoretical values. 

The liquid chromatography system was an Agilent 1260 Infinity II HPLC (Agilent, Santa Clara, CA, USA) containing a 1260 Infinity II Quaternary Pump (model G7111A), a 1260 Infinity II auto-sampler (model G7129A), a 1260 Infinity II Multicolumn Thermostat (model G7116A), and a 1260 Infinity II Diode Array Detector (model G7115A). The data were developed by employing the software Agilent OpenLAB CDS LC ChemStation. The separation was achieved using a Poroshell 120 EC-C18 (150 × 4.6 mm i.d., particle size 4 μm; Agilent, Santa Clara, CA, USA), kept at 20 °C. The samples were run using 20% water (A) and 80% acetonitrile (B), enriched with trifluoroacetic acid (0.1% *v*/*v*) in isocratic elution for 30 min. The flow rate was 0.8 mL/min. The UV detector was set at 254 nm. 

For the high-resolution mass spectrometry analysis, 10 μg/mL of each investigated compound was dissolved in ACN/H_2_O 80/20 with 0.1% of formic acid and injected into the MS spectrometer through a syringe pump at a flow rate of 5 μL/min. A Thermo Fischer Orbitrap FusionTM TribridTM, working in MS scan in an *m*/*z* range of 80 to 500 *m*/*z* by utilizing the Orbitrap as the detector type at 240,000 of mass resolution (FWHM), was used. **CD1-3** spectra were recorded in the positive ion mode.

### 3.1. Chemistry

#### 3.1.1. Synthesis of (S)-5-isopropyl-2-methyl-phenyl 2-(4-isobutilphenyl) Propanoate (**CD1**)

Ibuprofen (1.44 mmol) was solubilized in 6 mL of N, N-dimethylformamide (DMF), 6 mL of dichloromethane (DCM), and 6 mL of triethylamine (TEA). Then, 1.1 eq mmol of BOP (1.58 mmol) was added to the solution refrigerated in an ice-water bath and kept under stirring for 30′. Similarly, another solution of Car (1 eq, 1.44 mmol), DCM (3 mL), and TEA (1.8 mL) was kept under stirring for 30′. The solution containing Car was added to that containing ibuprofen, and the reaction was followed by 4 h at 40 °C. After the removal of solvents under reduced pressure, 10 mL of H_2_O was added to the residue, which was washed three times with AcOEt (20 mL). The organic phases were treated sequentially with 1 M HCl, H_2_O, 1 M NaHCO_3_, and H_2_O, and then they were collected and evaporated. The crude product after chromatography (eluent: hexane/ethyl acetate, 9.5/0.5) was obtained as an oil.

(S)-5-isopropyl-2-methylphenyl 2-(4-isobutilphenyl) propanoate **(CD1)**. Yield: 22%; Rf = 0.79, hexane/ethyl acetate, 9.5/0.5; ^1^H NMR (300 MHz, CDCl_3_) δ: 0.99 (6H, d, J = 6.9 Hz), 1.29 (6H, d, J = 6.9 Hz), 1.73 (3H, d, J = 7.2 Hz), 1.92 (3H, s), 1.94 (1H, m), 2.54 (2H, d, J = 6.9 Hz), 2.91 (1H, m), 4.05 (1H, q), 6.89 (1H, s), 7.05 (1H, d, J = 7.8 Hz), 7.12 (1H, d, J = 8.1 Hz), 7.23 (2H, d, J = 7.5 Hz), 7.40 (2H, d, J = 7.8 Hz); ^13^C NMR (75 MHz, CDCl_3_) δ: 15.47 (CH_3_), 18.25 (CH_3_), 22.39 (CH_3_), 22.43 (CH_3_), 23.97 (CH_3_), 24.01 (CH_3_), 30.31 (CH), 33.64 (CH), 45.14 (CH_2_), 45.36 (CH), 119.79 (CH), 123.99 (CH), 127.33 (C), 127.53 (2 × CH), 129.51 (2 x CH), 130.92 (CH), 137.46 (C), 140.88 (C), 147.93 (C), 149.34 (C), 172,89 (CO). Calcd for C_23_H_30_O_2_: C, 81.61; H, 8.93; O, 9.45. Found: C, 81.50; H, 8.89; O, 9.60. HR-MS (ESI) *m/z*: [M + H]^+^ = 339.2310.

#### 3.1.2. General Procedures for the Synthesis of **CD2** and **CD3**

**CD2** and **CD3** were synthesized using flurbiprofen or naproxen (1 eq), respectively, and dissolved in tetrahydrofuran (THF) (20 mL) prior to the addition of DCC (1 eq), and the mixture was stirred for 1 h at rt. Then, Car (1 eq) and DMAP (0.033 eq) were added, and the mixture was stirred for 24 h at rt. After the filtration and evaporation of the solvent, DCM was added, and the solution was treated with NaHCO_3_ and dried over Na_2_SO_4_. After the evaporation of the DCM and the purification on the silica gel with DCM as the eluent, the Car derivatives were obtained as oils.

(R)-5-isopropyl-2-methyl-phenyl-2-(6-methoxynaphthalen-2-yl) propanoate (**CD2**). Yield: 78%; Rf = 0.78, CH_2_Cl_2_; ^1^H NMR (300 MHz, CDCl_3_) δ: 1.28 (6H, d, J = 7.2 Hz), 1.75 (3H, d, J = 7.2 Hz), 2.04 (3H, s), 2.93 (1H, m), 4.09 (1H, q), 6.92 (1H, s), 7.08 (1H, d, J = 9.3 Hz), 7.16 (1H, d, J = 7.8 Hz), 7.35 (2H, m), 7.46 (1H, m), 7.54 (3H, m), 7.65 (2H, d, J = 9.9 Hz); ^13^C NMR (75 MHz, CDCl_3_) δ: 15.61 (CH_3_), 18.36 (CH_3_), 23.94 (2 × CH_3_), 33.61 (CH), 45.19 (CH), 115.40 (CH), 115.71 (CH), 119.64 (CH), 123.85 (CH), 124.17 (CH), 127.15 (CH), 127.79 (CH), 128.54 (CH), 128.99 (2 x CH), 130.99 (CH), 135.49 (C), 141.37 (C), 141.47 (C), 148.09 (C), 149.18 (C), 158.18 (C), 161.47 (C), 172.18 (CO). Calcd for C_25_H_25_FO_2_: C, 79.76; H, 6.69; F, 5.05; O, 8.50. Found: C, 79.74; H, 6.65; F, 5.07; O, 8.54. HR-MS (ESI) *m/z*: [M + H]^+^ = 377.1900.

(S)-5-isopropyl-2-methyl-phenyl-2-(6-methoxynaphthalen-2-yl) propanoate (**CD3**). Yield: 15%; Rf = 0.86, CH_2_Cl_2_; ^1^H NMR (300 MHz, CDCl_3_) δ: 1.27 (6 H, d, J = 7.2 Hz), 1.81 (3H, d, J = 7.2 Hz), 1.94 (3H, s), 2.89 (1H, m), 3.95 (3H, s), 4.18 (1H, q), 6.88 (1H, s), 7.04 (1H, d, J = 9.3 Hz), 7.11 (1H, d, J = 7.8 Hz), 7.25 (2H, m), 7.62 (1H, d, J = 9.9 Hz), 7.77 (2H, t), 7.87 (1H, s). ^13^C NMR (75 MHz, CDCl_3_) δ: 15.61 (CH_3_), 18.52 (CH_3_), 23.93 (CH_3_), 23.97 (CH_3_), 33.60 (CH), 45.63 (CH), 55.31 (CH_3_), 105.70 (CH), 119.15 (CH), 119.73 (CH), 124.02 (CH), 126.37 (CH), 127.29 (C), 127.35 (CH), 129.06 (2 x CH), 129.39 (CH), 130.92 (C), 133.94 (C), 135.28 (C), 147.99 (C), 149.30 (C), 157.81 (C), 172.89 (CO). Calcd for C_24_H_26_O_3_: C, 79.53; H, 7.23; O, 13.24. Found: C, 79.48; H, 7.25; O, 13.27. HR-MS (ESI) *m/z*: [M + H]^+^ = 363.1947.

### 3.2. In Silico Evaluation of Physicochemical Properties

The prediction of physicochemical properties for **CD1-3** was performed through the online SwissADME program (online access: 20 April 2022 at https://swissadme.ch) to obtain relative results of physicochemical parameters (water solubility, logP, MW, polar surface area, number of HBD and HBA, and number of rotary bonds) [37].

### 3.3. Stability in Gastrointestinal Fluids

The SGF and SIF fluids, respectively, were arranged according to USP requirements. The stock solutions containing **CD1-3** were added up to preheated SGF and SIF and placed in a 37 °C shaking water bath. At programmed time points—0, 15, 30, and 60′ for SGF and 0, 60, 120, and 180′ for SIF—100 μL was deproteinized with 100 μL of ice-cold ACN containing 0.5% *v/v* of formic acid and positioned into micro-centrifuge tubes. The samples were centrifuged at 4 °C and 12,000 rpm for 10′. After filtration, the supernatant was evaluated by HPLC.

### 3.4. Human Plasma Stability

Human plasma was acquired from 3H Biomedical (Uppsala, Sweden, Europe). Initially, a stock solution containing **CD1-3** was treated with 0.02 M phosphate buffer (pH 7.4) to give a final volume of 1 mL (80% plasma), and then it was added to a pre-heated (37 °C) plasma fraction to stop enzymatic hydrolysis. Samples of 100 μL were taken at various times, and 200 μL of 0.01 M HCl in MeOH was used to stop the enzymatic activity. After centrifugation for 5 min at 5000× *g*, the supernatant was examined by HPLC.

### 3.5. Biological Assays

#### 3.5.1. Cell Cultures

##### 3T3-L1

Mouse embryonic 3T3-L1 cells, purchased from ATCC (Manassas, VA, USA), were cultured in low-glucose (1 g/L) D-MEM medium (Dulbecco’s modified Eagle’s medium) (Lonza) supplemented with 10% FBS, L-glutamine 0.584 g/L, and 1% penicillin/streptomycin at 37 °C in 5% CO_2_.

##### WJ-MSC

The mesenchymal stem cells WJ-MSCs were extracted from human umbilical cords of full-term births. After the removal of blood vessels, the Wharton’s jelly was finely cut, washed with serum-free (D-MEM) (Lonza), and centrifuged for 5 min at 250× *g* at rt. The pellets obtained were first treated with collagenase type IV (2 mg/mL) (Sigma, Merck Millipore, Darmstadt, Germany) for 18 h at 37 °C and then with 2.5% trypsin-EDTA (GIBCO) for 30′ at 37 °C, under stirring. The cells obtained from two subcultures, WJ-1 and WJ-2, were washed in PBS and cultured in hMSC (Human Mesenchymal Stem Cells) growth medium (Lonza) in 5% CO_2_ at 37 °C for 3–4 days until reaching confluence. Institutional Review Board approval for the cell culture was received for all experiments from the Institutional Committee for Human Experimentation of Chieti Hospital No. 1879/09COET. This approval concerns the authorization to harvest the umbilical cord to obtain mesenchymal stem cells (WJ-MSCs) and their use only for in vitro research.

##### THP-1

The human monocytic cell line THP-1 (American Type Culture Collection, Manassas, Va.) was grown in RPMI 1640 (Sigma–Aldrich; Merck Millipore, Darmstadt, Germany) medium supplemented with 2 mM L-glutamine, 1% penicillin/streptomycin, and 10% FBS at 37 °C in a humidified atmosphere of 5% CO_2_.

#### 3.5.2. Adipogenic Differentiation

3T3-L1 and WJ-MSCs cells were grown to 70–80% confluence in D-MEM supplemented with 10% FBS, 0.584 g/L glutamine, 5 mM glucose, 5 mM sodium acetate, and 1% penicillin/streptomycin. The medium was changed every 48 h, and the viability was checked with trypan blue. At approximately 80% confluence, differentiation was induced in a selective medium (glucose D-MEM supplemented with 10% FCS, 1.7 mol/L insulin, 0.5 mM 3-isobutyl-1-methylxanthine (IBMX), 1 μM dexamethasone, and 1% penicillin/streptomycin) for 5 and 7 days in 3T3-L1 and for 17 days in WJ-MSCs, until complete differentiation.

The differentiation of the different cell cultures in the presence of 25 μM Car, NSAIDs (ibuprofen, flurbiprofen, or naproxen), the physical mixture of selected NSAIDs and 25 μM Car, and CD1–3 was evaluated. The differentiation test, as lipid accumulation in adipocytes, was revealed by oil red-O staining. The expression of the transcription factor ChREBP was evaluated with western blotting.

#### 3.5.3. Oil-Red O Staining to Detect Adipogenic Differentiation

The intracellular lipid accumulation in mature adipocytes, during adipogenesis, was determined by Oil-Rd O staining (Sigma-Aldrich Co., St. Louis, MO, USA) in 3T3-L1 at 5 and 7 days and in WJ-MSC at 17 days of differentiation. The differentiated cells were fixed in 4% formaldehyde in PBS for 10′, washed with 60% isopropanol (2-PrOH), and stained with 0.2% Oil-Red O in 60% 2-PrOH for 10′. To remove the unincorporated dye, the cells were washed many times with H_2_O and destained in 60% 2-PrOH for 15′. The red-stained cells were observed with an optical Zeiss microscope and photographed. Lipid quantification was determined by dissolving stained cellular oil droplets in 60% 2-PrOH and quantified spectrophotometrically at 580 nm.

#### 3.5.4. Induction of Cell Differentiation (Stimulation of Transformation into Tissue Macrophages)

The induction of cell differentiation was performed upon reaching 80% confluence. THP-1 cells (~2 × 10^5^ cells/mL) were plated as a control group w/o treatment, treated overnight with 100 ng/mL Car, NSAIDs (ibuprofen, flurbiprofen, or naproxen), and **CD1–3**, or supplemented with 100 ng/mL lipopolysaccharide (LPS derived from *E. coli* purchased from Sigma–Aldrich, Saint Louis, MO, USA). The cells then were collected by centrifuging for 8′ at 1000 rpm, and cell extracts were made according to standard procedures. For macrophage differentiation, the THP-1 cells (~2 × 10^5^/mL) were incubated with 100 ng/mL PMA (Phorbol myristate acetate) (Sigma–Aldrich, Saint Louis, MO, USA) for 3 days at 37 °C. After removing the PMA-containing media, the cells were incubated for a further 4 days in a conventional medium that was treated earlier with 25 μL of each compound alone or supplemented with 100 ng/mL LPS, according to a specific time-course. THP-1-derived macrophages were detached by Trypsin-EDTA 1X (Euro Clone) treatment for 10′ at 37 °C. Differentiated cells were counted using the vital dye Trypan–blue solution (0.4%) (Sigma–Aldrich, Saint Louis, MO, USA) in a Burker chamber and analyzed for viability [42]. The cells were then lysed to obtain a sample containing both intracellular and released cytokines in the cell culture. 

#### 3.5.5. ELISA

TNF-α levels were measured in cell culture supernatants using a Quantikine solid-phase ELISA kit (R&D System, MN, USA), following the manufacturer’s instructions. The plate was read in a microplate reader (GloMax^®^ Multi Detection System, Promega, MI, Italy) at 450 nm. TNF-α levels (pg/mL) were obtained based on the calibration curves prepared with the cytokine standard obtained by the supplier. The intra- and inter-assay reproducibility was >90%. The minimum detectable (MDD) was 4.8 pg/mL.

#### 3.5.6. Western Blotting

The cells were incubated on ice for 20′ in a lysis buffer containing 50 mM Tris^.^Cl pH 7.6, 1% Triton X100, 0.1% SDS, 250 mM NaCl, 5 mM EDTA, proteases, and phosphatases inhibitor cocktail (Thermo Fisher Scientific, Waltham, MA, USA). The samples were then centrifuged at full speed in a refrigerated microfuge, and the supernatant was recovered and assayed for protein content using the Bradford assay (Bio-Rad Laboratories, Milan, Italy). Protein extracts were run on precast 4–12% Bis-Tris protein gels (Invitrogen, Thermo Fisher Scientific, Waltham, MA, USA) and transferred onto PVDF Stacks (Invitrogen, Thermo Fisher Scientific, Waltham, MA, USA) through the iBlot 2 semidry apparatus (Invitrogen, Thermo Fisher Scientific, Waltham, MA, USA). The membranes were then stained with Ponceau Red (Sigma-Aldrich, Milano, Italy) to verify the proper protein transfer and blocked at rt for 1 h with 5% non-fat dry milk in TBST containing 0.1% Tween20. The protein level was analyzed by western blotting by incubating the membranes O/N at 4 °C with an anti-ChREBP (1:100) (Santa Cruz Biotechnology, Heidelberg, Germany) antibody diluted in 5% non-fat milk in TBST 0.1% Tween20. The membranes were then washed three times for 10′ with TBST and incubated for 1 h at rt with anti-rabbit HRP-conjugated secondary antibody (Santa Cruz Biotechnology, Heidelberg, Germany) diluted 1/2000 in TBST containing 5% non-fat milk. The membranes were washed three times for 10′, incubated in Super Signal West Pico (Thermo Fisher Scientific Inc, Pierce Biotechnology, Rockford, IL, USA) chemiluminescent substrate, and detected using a ChemiDoc XRSplus imaging system (Bio-Rad Laboratories, Milano, Italy). The optical densities of the blot bands were ultimately revealed by employing a computer-assisted densitometer (ImageJ, U.S. National Institutes of Health, Bethesda, MD, USA) and normalized versus the β-actin housekeeping protein internal control.

## 4. Conclusions

To date, many scientific efforts have been made to understand the complex mechanism underlying obesity. However, no effective anti-obesity drugs have been discovered. 

In this study, **CD1** and **CD3** were found to have an inhibitory effect on lipid accumulation in both 3T3-L1 and WJ-MSCs cells, but **CD3**—obtained by a direct linkage between carvacrol and naproxen—showed additional pharmacological and drug-like properties. Derivative **CD3**, endowed with the best physicochemical properties and stability, was able to neutralize the inflammatory effect of LPS through a decrease in TNF-α levels and reduced the expression levels of ChREBP, which is the major factor responsible for de novo lipogenesis. In the future, in vivo studies are required to confirm the anti-obesity effects of Car derivatives—especially **CD3**—and gain new insight into the mechanism by which **CD3** reduces the adipogenesis of 3T2-L1 and WJ-MSCs preadipocytes and TNF-α-induced inflammation in THP-1 cells.

## Figures and Tables

**Table 1:** In silico evaluation of **CD1-3** physicochemical properties ^a^.

Compounds	Water Solubility (mg/mL)	Lipinski’s Rule				TPSA	MR
		MW	LogP	HBA	HBD		
**CD1**	1.76 × 10^−5^	338.48	5.90	2	0	26.30	106.17
**CD2**	1.40 × 10^−5^	376.46	6.35	3	0	26.30	112.17
**CD3**	2.24 × 10^−5^	362.46	5.60	3	0	35.53	110.78
**Car**	3.79 × 10^−2^	150.22	2.82	1	1	20.23	48.01

^a^ Prediction SwissADME platforms. Abbreviations: MW = Molecular weight, LogP = water/octanol partition coefficient, HBA = Hydrogen Bond Acceptors, HBD = Hydrogen Bond Donors, TSPA = Topological Polar Surface Area, MR = Molar Refractivity.

**Table 2:** Kinetic data for the hydrolysis of **CD1-3** at 37 °C ^a^.

		CD1		CD2		CD3	
		t_½_ (h)	k_obs_ (h^−1^)	t_½_ (h)	k_obs_ (h^−1^)	t_½_ (h)	k_obs_ (h^−1^)
Chemical hydrolysis	pH 1.2	12.89 (±1.2)	0.054 (±0.01)	13.5 (±1.2)	0.051 (±0.002)	16.92 (±2.1)	0.041 (±0.001)
	pH 7.4	5.93 (±0.5)	0.117 (±0.012)	8.92 (±0.9)	0.078 (±0.002)	9.76 (±1.5)	0.071 (±0.001)
Human plasma		2.53 (±0.21)	0.274 (±0.017)	3.41 (±0.1)	0.203 (±0.012)	4.97 (±0.5)	0.139 (±0.031)

^a^ Number of experiments: three; standard deviation is given in parentheses. Abbreviations: t_1/2_ = the time required for the concentration of a drug to decrease by 50%; K_obs_ = the observed rate constant is a linear function of the drug concentration indicating that the reaction is first-order in metabolites.

## Data Availability

Not applicable.

## Supplementary Materials

The following supporting information can be downloaded at: https://www.mdpi.com/article/10.3390/ph16030340/s1, HPLC chromatograms and ^1^H-, ^13^C-NMR, and HR-MS spectra of **CD1-3**; Figure S1: Reduction in adipocyte differentiation by Car in 3T3-L1 cells; Figure S2: Reduction in adipocyte differentiation by Car in WJ-MSCs cells.
