# Supplementary material for: Anti-Obesity and Anti-Inflammatory Effects of Novel Carvacrol Derivatives on 3T3-L1 and WJ-MSCs Cells
Source: Pharmaceuticals (Basel). 2023 Feb 22;16(3):340. doi: 10.3390/ph16030340 (PMC10055808; doi:10.3390/ph16030340)
Supplement: Supplementary file 1 [file pharmaceuticals-16-00340-s001.zip › pharmaceuticals-2115560-supplementary.pdf]

HPLC CHROMATOGRAMS

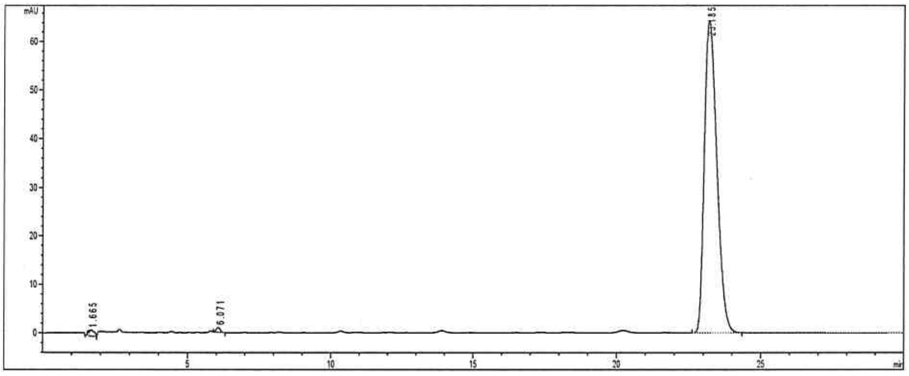

Compound	Retention time	Area%
CD1	23.19	>98

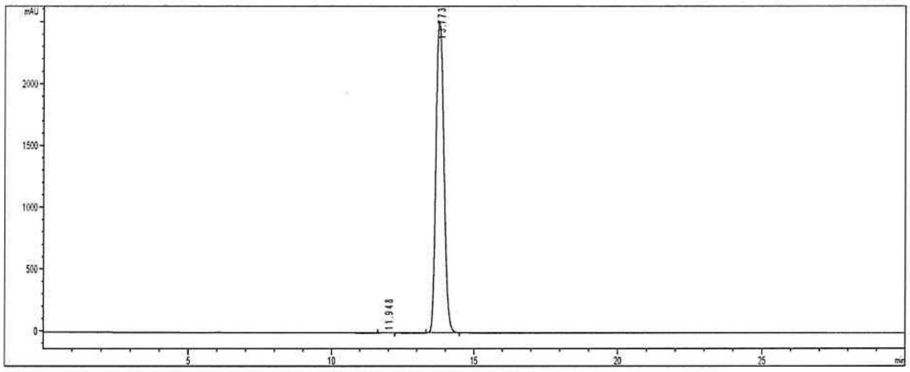

Compound	Retention time	Area%
CD2	13.77	>99

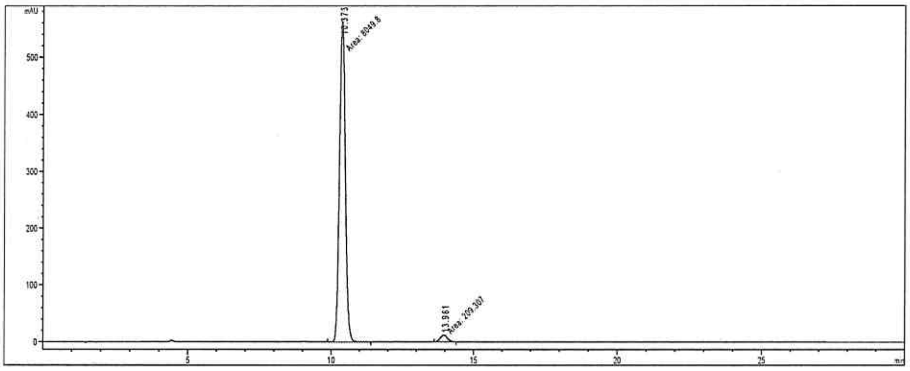

Compound	Retention time	Area%
CD3	10.40	>97

CDI

Agilent Technologies

Sample Name:
Ibu-car_fraz_27-36
Data Collected on:
m300-mercury300
Archive directory:
/home/caccia/vnmrsys/data
Sample directory:
OLP-RA_MARI1_20211021_01
FidFile: PROTON

Pulse Sequence: PROTON (s2pul)
Solvent: cdcl3
Data collected on: Feb 17 2022

Operator: caccia

Relax. delay 1.000 sec
Pulse 45.0 degrees
Acq. time 1.706 sec
Width 4803.1 Hz
32 repetitions
OBSERVE H1, 300.1976543 MHz
DATA PROCESSING
FT size 16384
Total time 1 min 29 sec

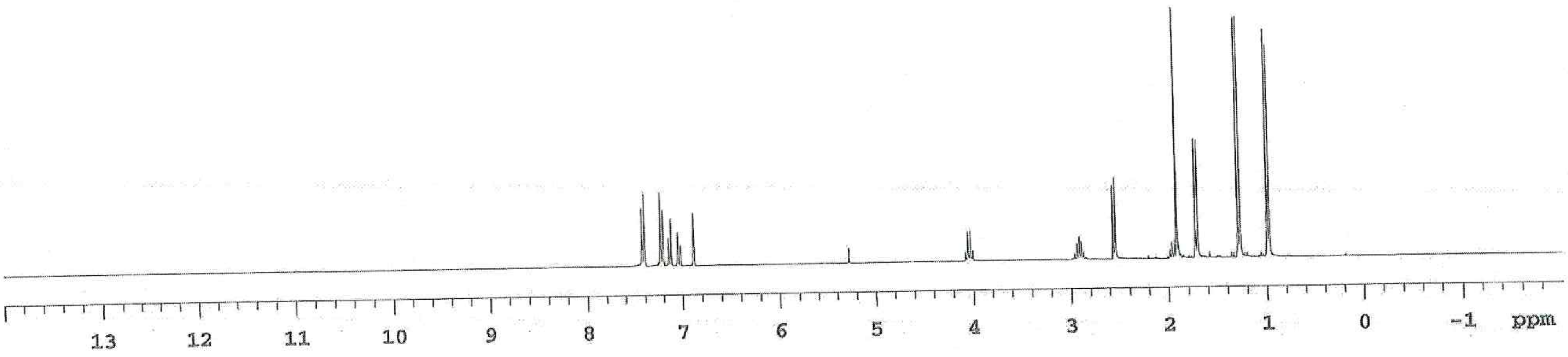

CD1

Agilent Technologies

Sample Name:
  Ibu-car_fraz_27-36
Data Collected on:
  m300-mercury300
Archive directory:
  /home/caccia/vnmrsys/data
Sample directory:
  OLP-RA_MARI1_20211021_01
FidFile: CARBON

Pulse Sequence: CARBON (s2pul)
Solvent: cdcl3
Data collected on: Feb 16 2022

Operator: caccia

Relax. delay 1.000 sec
Pulse 45.0 degrees
Acq. time 0.868 sec
Width 18867.9 Hz
2000 repetitions
OBSERVE C13, 75.4847602 MHz
DECOUPLE H1, 300.1991980 MHz
Power 38 dB
continuously on
WALTZ-16 modulated
DATA PROCESSING
Line broadening 0.5 Hz
FT size 32768
Total time 1 hr, 4 min

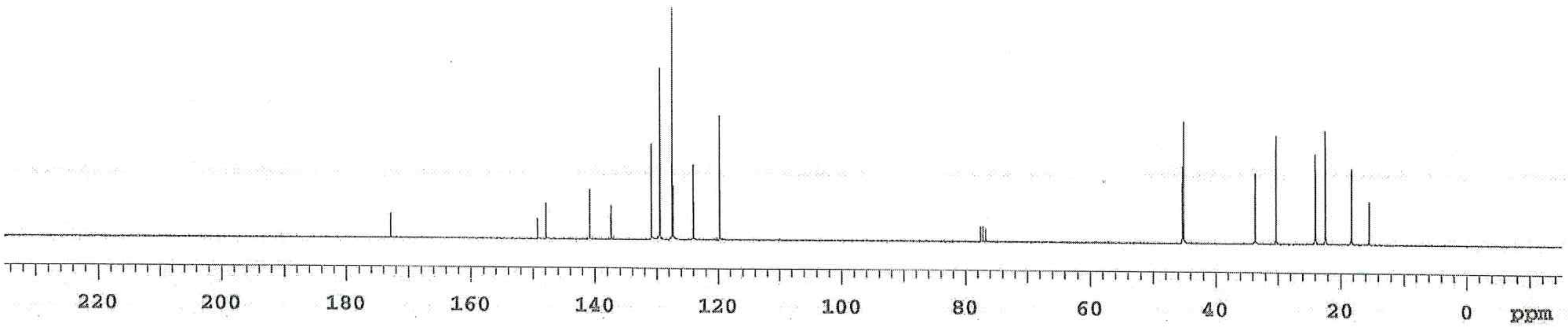

CD2

Sample Name:
Flu-car_fraz_39-71
Data Collected on:
m300-mercury300
Archive directory:
/home/caccia/vnmrsys/data
Sample directory:
OLP-RA_MARI1_20211021_01
FidFile: PROTON

Pulse Sequence: PROTON (s2pul)
Solvent: cdcl3
Data collected on: Feb 15 2022

Operator: caccia

Relax. delay 1.000 sec
Pulse 45.0 degrees
Acq. time 1.706 sec
Width 4803.1 Hz
32 repetitions
OBSERVE H1, 300.1976543 MHz
DATA PROCESSING
FT size 16384
Total time 1 min 29 sec

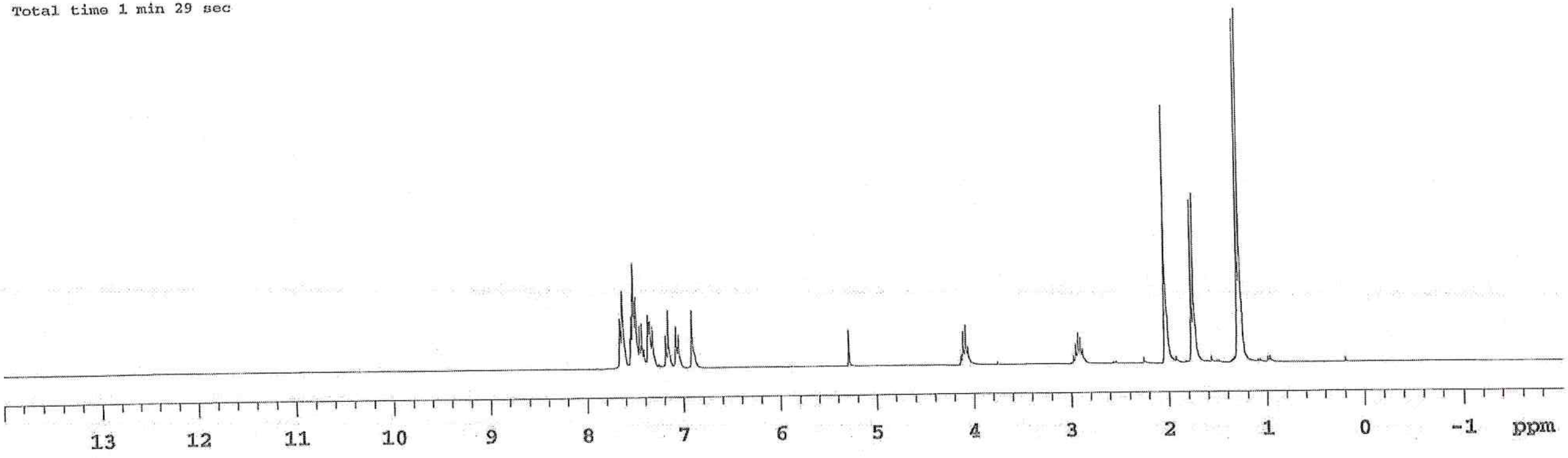

CD2

Agilent Technologies

Sample Name:
Flur-car_fraz_39-71
Data Collected on:
m300-mercury300
Archive directory:
/home/caccia/vnmrsys/data
Sample directory:
OLP-RA_MARI1_20211021_01
FidFile: CARBON

Pulse Sequence: CARBON (s2pul)
Solvent: cdcl3
Data collected on: Feb 16 2022

Operator: caccia

Relax. delay 1.000 sec
Pulse 45.0 degrees
Acq. time 0.868 sec
Width 18867.9 Hz
2000 repetitions
OBSERVE C13, 75.4847602 MHz
DECOUPLE H1, 300.1991980 MHz
Power 38 dB
continuously on
WALTZ-16 modulated
DATA PROCESSING
Line broadening 0.5 Hz
FT size 32768
Total time 1 hr, 4 min

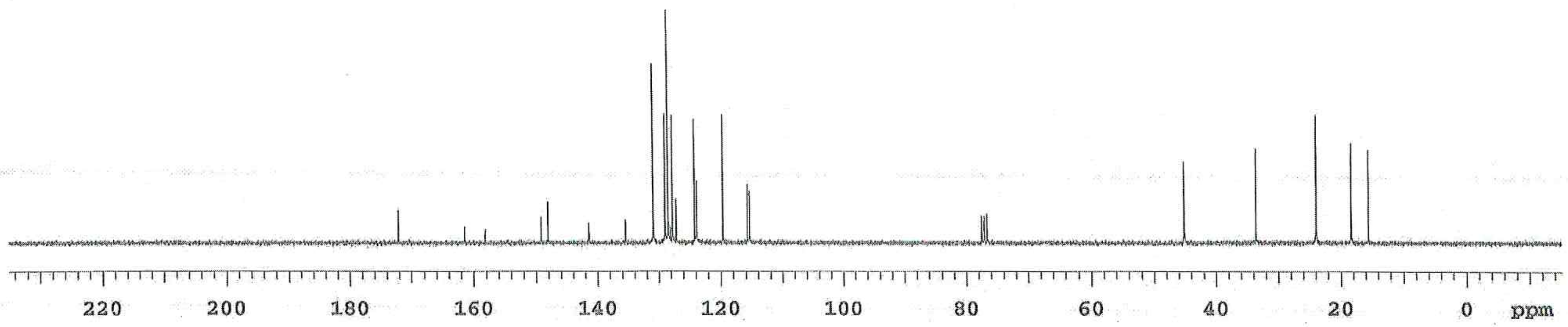

CD3

Sample Name:
Naprox-CAR
Data Collected on:
m300-mercury300
Archive directory:
/home/caccia/vnmrsys/data
Sample directory:
OLP-RA_MARI1_20211021_01
FidFile: PROTON

Pulse Sequence: PROTON (s2pul)
Solvent: cdcl3
Data collected on: Feb 15 2022

Operator: caccia

Relax. delay 1.000 sec
Pulse 45.0 degrees
Acq. time 1.706 sec
Width 4803.1 Hz
32 repetitions
OBSERVE H1, 300.1976543 MHz
DATA PROCESSING
FT size 16384
Total time 1 min 29 sec

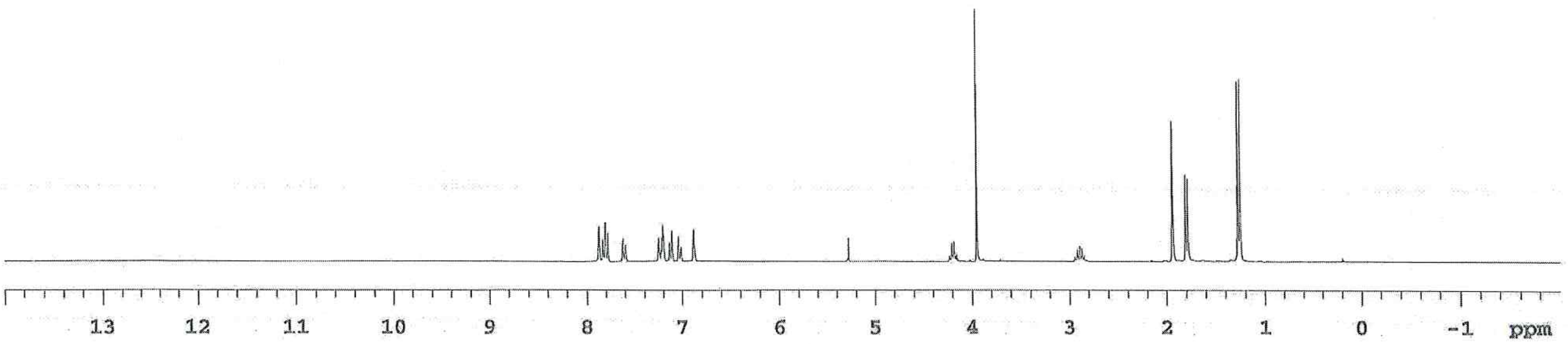

CD3

Agilent Technologies

Sample Name:
Naprox-CAR
Data Collected on:
m300-mercury300
Archive directory:
/home/caccia/vnmrsys/data
Sample directory:
OLP-RA_MARI1_20211021_01
FidFile: CARBON

Pulse Sequence: CARBON (s2pul)
Solvent: cdcl3
Data collected on: Feb 15 2022

Operator: caccia

Relax. delay 1.000 sec
Pulse 45.0 degrees
Acq. time 0.868 sec
Width 18867.9 Hz
1664 repetitions
OBSERVE C13, 75.4847602 MHz
DECOUPLE H1, 300.1991980 MHz
Power 38 dB
continuously on
WALTZ-16 modulated
DATA PROCESSING
Line broadening 0.5 Hz
FT size 32768
Total time 1 hr, 4 min

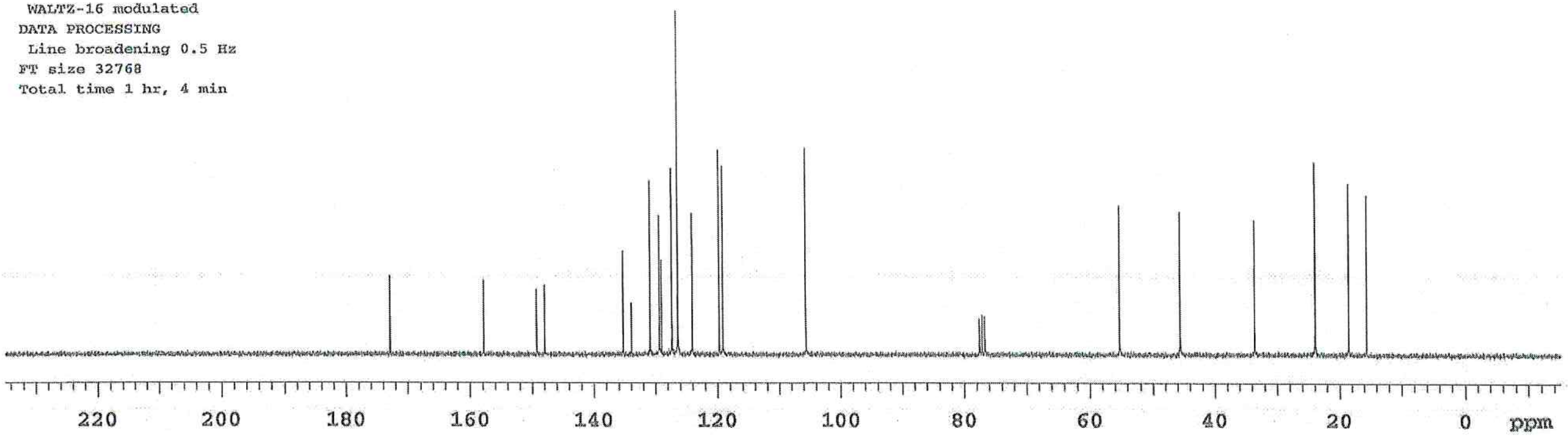

HR-MS SPECTRA

High Resolution Mass Spectrometry Analysis

10 µg/mL of each investigated compound was dissolved in ACN/H2O 80/20 with 0.1% of formic acid and injected into the mass spectrometer through a syringe pump at a flow rate of 5 ul/min. The mass spectrometer was a Thermo Fischer Orbitrap FusionTM TribridTM operating in MS scan in the m/z range of 80 to 500 m/z by using the Orbitrap as detector type at 240,000 of mass resolution (FWHM). All compounds were acquired in positive ion mode.

CD1

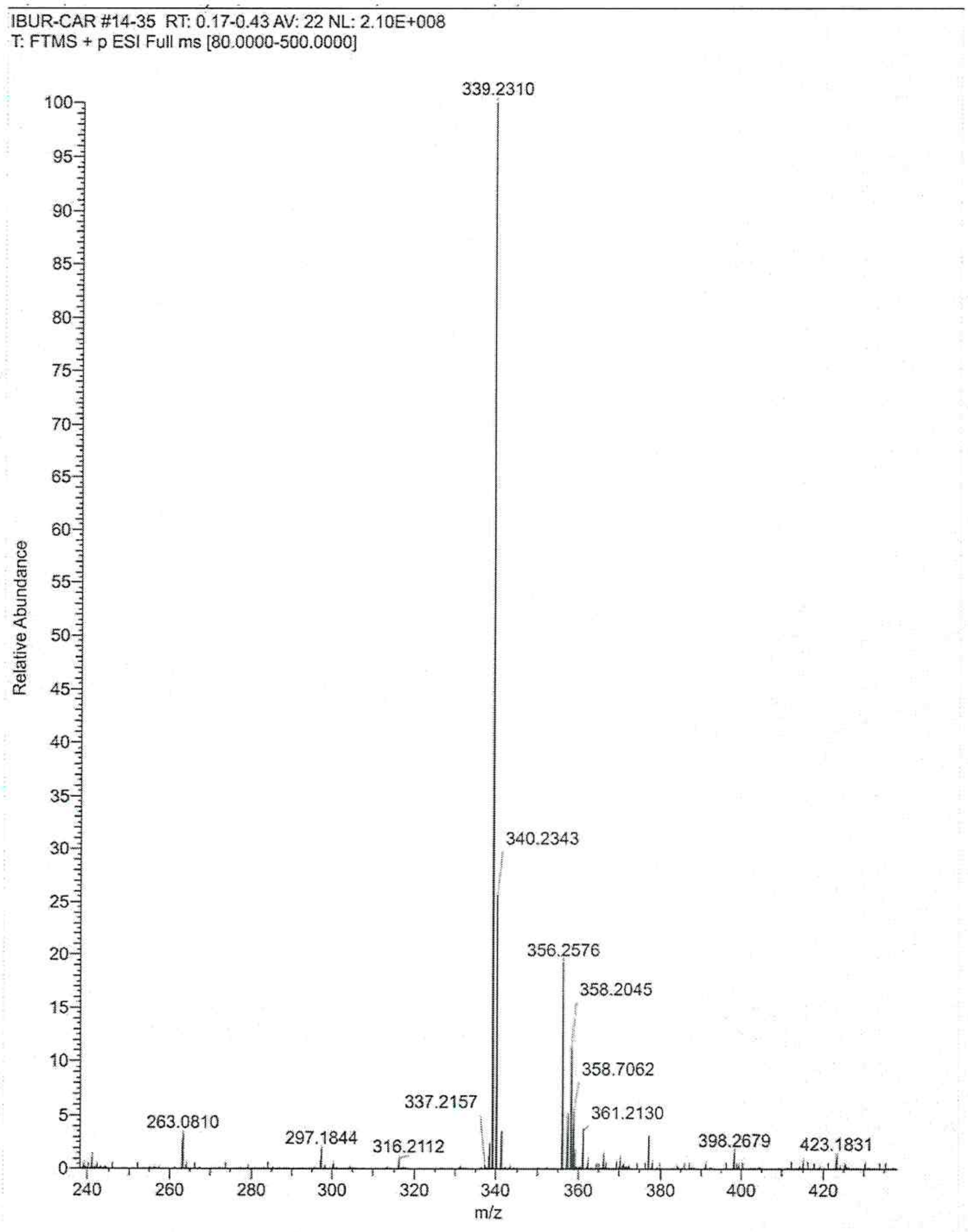

CD2

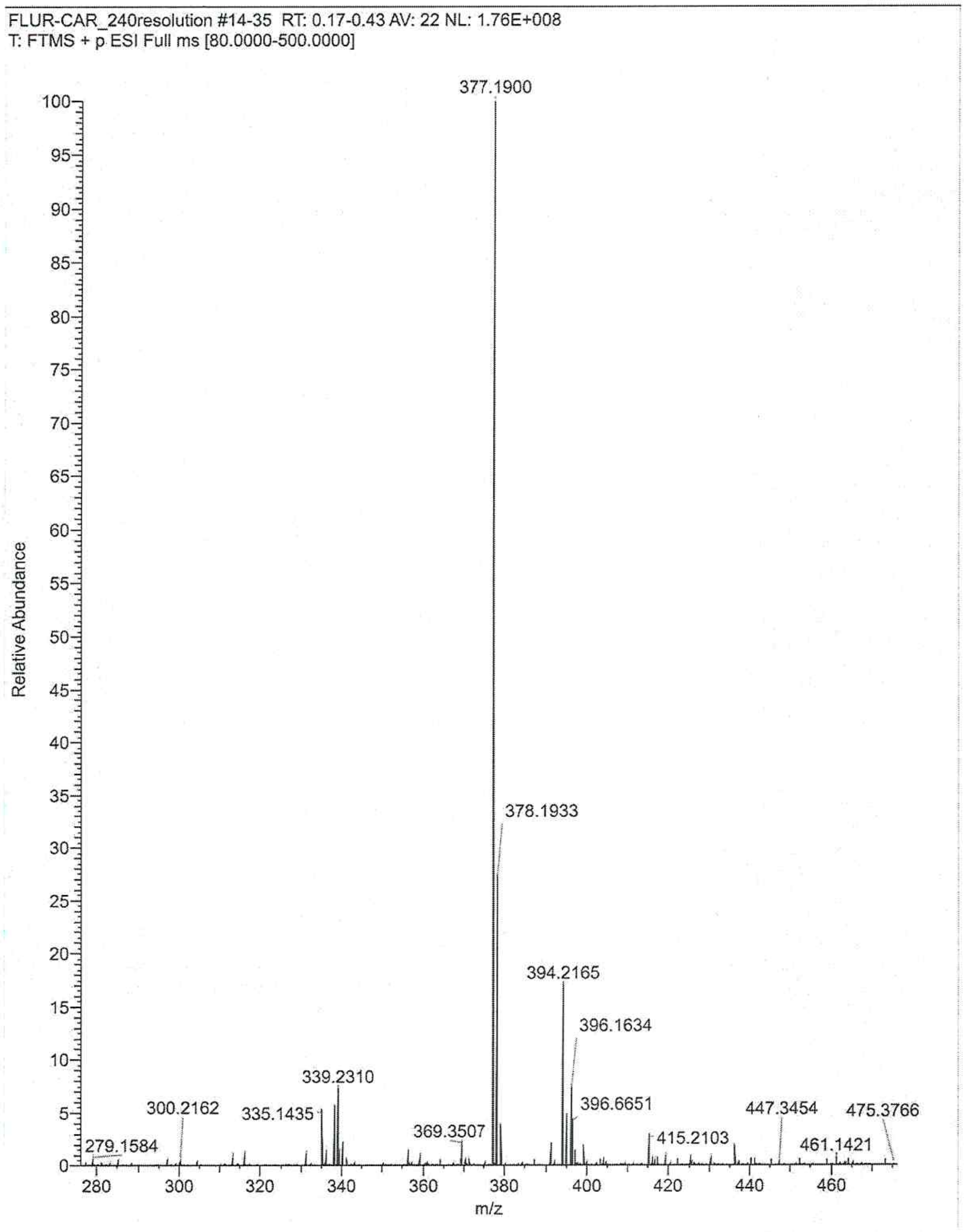

FLUR-CAR_240resolution #14-35 RT: 0.17-0.43 AV: 22 NL: 1.76E+008
T: FTMS + p ESI Full ms [80.0000-500.0000]
377.1900
378.1933
394.2165
396.1634
396.6651
339.2310
300.2162
335.1435
369.3507
279.1584
415.2103
447.3454
475.3766
461.1421
Relative Abundance
m/z

CD3

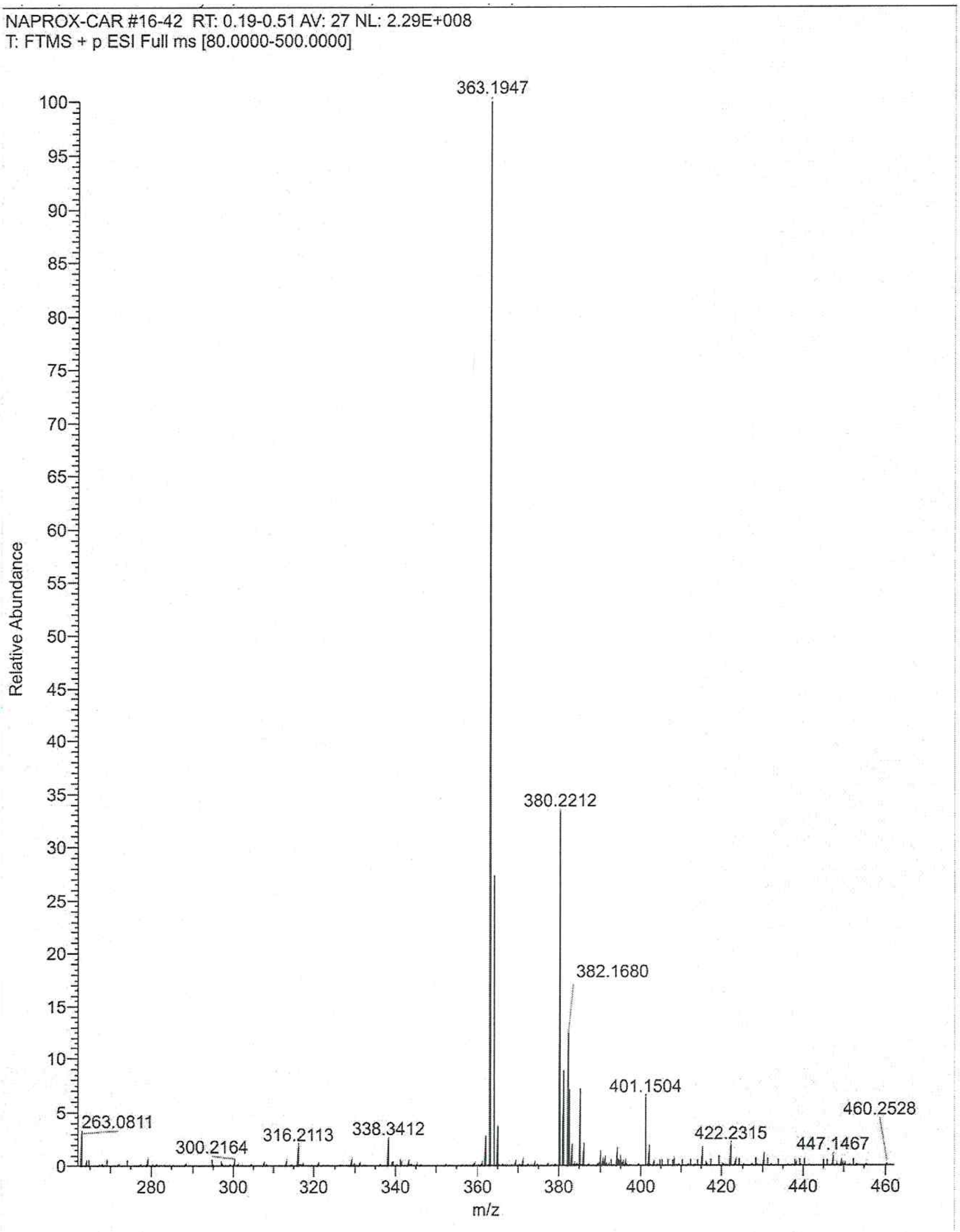
NAPROX-CAR #16-42 RT: 0.19-0.51 AV: 27 NL: 2.29E+008
T: FTMS + p ESI Full ms [80.0000-500.0000]
363.1947
380.2212
382.1680
401.1504
460.2528
422.2315
447.1467
263.0811
300.2164
316.2113
338.3412
Relative Abundance
m/z

Additional biological data

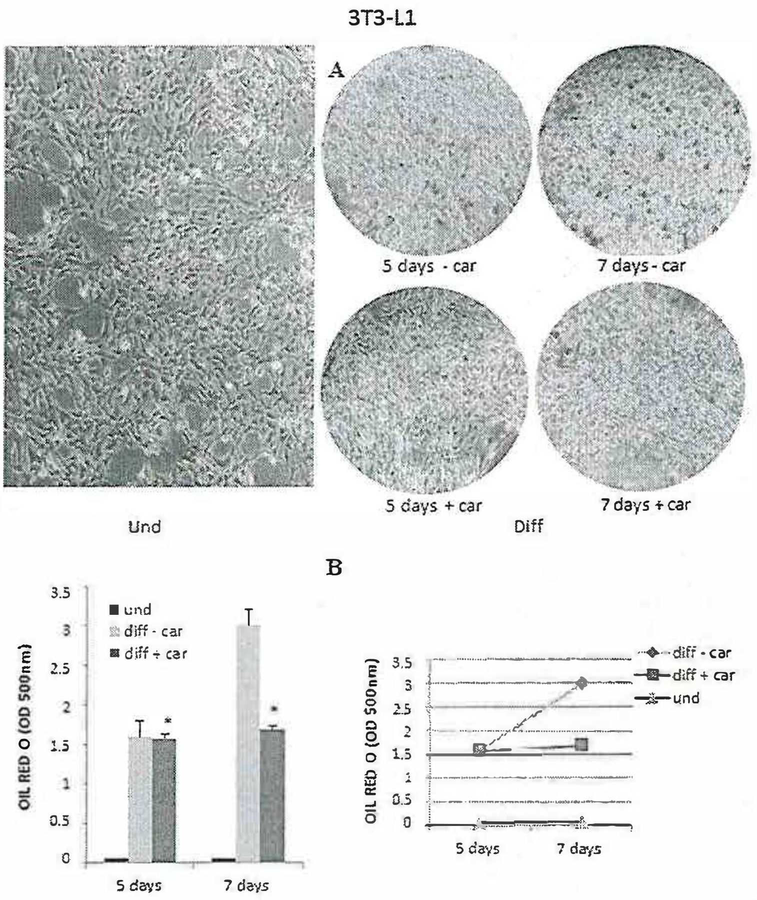

Figure S1. Reduction of adipocyte differentiation by car in 3T3-L1 cells. 3T3–L1 preadipocytes were grown in adipogenic differentiation medium until total differentiation (7 days) and treated with 25 μM car. The visualization of triglyceride accumulation was conducted by Oil Red O staining (A). Lipid accumulation was measured through a spectrophotometer at 5 and 7 days of differentiation (B). Und = Undifferentiated cells; Diff = Differentiated cells without (diff - car) or with car (diff + car). Data are presented as mean ± SD ($n = 3$). *$p < 0.05$: treated cells vs control (diff - car) and car (diff + car).

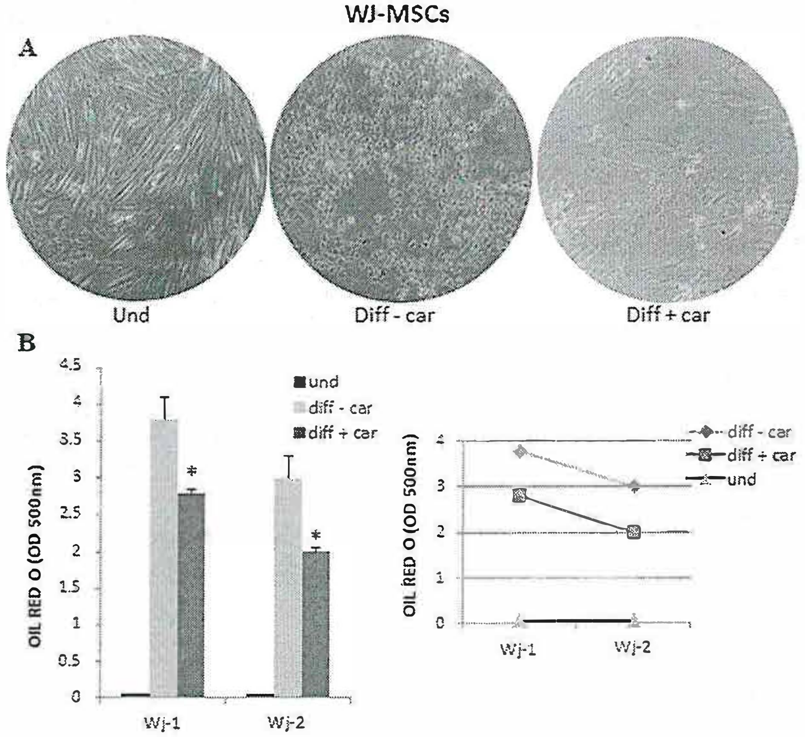

Figure S2. Reduction of adipocyte differentiation by car in WJ-MSCs cells. Two subcultures of Wharton's jelly WJ-MSCs, WJ-1, and WJ-2, were grown in an adipogenic differentiation medium until total differentiation (17 days) and treated with 25 µM car. The visualization of triglyceride accumulation was conducted by Oil Red O staining. Lipid accumulation was measured through a spectrophotometer. Und = Undifferentiated cells; Diff = Differentiated cells without (diff - car) or with car (diff + car). Data are presented as mean ± SD (*n* = 3). *$p < 0.05$: treated cells vs control (diff - car) and car (diff + car).
